# The ARDSnet protocol may be detrimental in COVID-19

**DOI:** 10.1186/s13054-020-03081-4

**Published:** 2020-06-16

**Authors:** Vasiliki Tsolaki, George E. Zakynthinos, Dimosthenis Makris

**Affiliations:** Intensive Care Unit, University Hospital of Larissa, University of Thessaly, Faculty of Medicine, Larissa, Greece

To the Editor,

Intensive care units are overwhelmed with COVID-19 ARDS patients during the last months, and increased mortality has been reported [1]. The Surviving Sepsis Campaign-COVID-19 guidelines and, recently, the American Thoracic Society (ATS) proposed to treat COVID-19 per ARDSnet protocol [2, 3]. However, there are a few issues we would like to address concerning the ventilatory strategy and fluid administration. Heart-lung interactions may play a crucial role, especially in the management of COVID-19 patients.

In the largest series of almost 1600 COVID-19 ICU patients, the median PaO_2_/FiO_2_ was 160 and the median PEEP used was 14 cmH_2_O [1]. It seems that PEEP was set according to predefined criteria (ARDSnet, SSC-COVID-19 guidelines, ATS statement) [2, 3]. However, COVID-19 ARDS does not seem to be “typical” [4]. In patients from our unit, the median static compliance was 52 ml/cmH_2_O, and this seems to be the case in most intubated patients in Greece (compliance of 50–65 ml/cmH_2_O, anecdotal reports) and other countries [4]. Ιn our patients, the mean PaO_2_/FiO_2_ value was 89. If we had followed the suggested protocols, we should have applied a PEEP of 18 cmH_2_O. Contrary, a median PEEP of 8 cmH_2_O was the “best” PEEP, evaluated combining respiratory variables (compliance, FRC, PaCO_2_) and hemodynamics through echocardiography (RV function, SPAP through tricuspid regurgitation). Trials of increased PEEP worsened hemodynamics and increased vasopressors. In most cases, fluid administration was decided considering inferior vena cava distensibility index and pulse pressure variation (tidal volume set at 8 ml/kg).

It is well-known that when lung compliance is relatively normal, even more than 50% of the alveolar pressure is transmitted to the pleural pressure. Relatively high PEEP (in a non-recruitable lung) may have a detrimental impact on hemodynamics, deteriorating venous return. Moreover, application of high PEEP when not-needed unnecessarily increases transpulmonary pressure forcing West’s zone 3 lung regions to zones 2 and 1, leading to dead space ventilation and increasing pulmonary vascular resistance [5]. Both effects are exacerbated in hypovolemic patients. Therefore, fluid restriction may not be so applicable in SARS-COV-2 ARDS. Hypovolemia and hemodynamic compromise in hypertensive patients might contribute to the observed increased mortality in those patients receiving diuretics as standard treatment, as hemodynamic instability leads to organ hypoperfusion and ultimately fatal multiorgan failure [1].

It seems that in most SARS-CoV-2 patients, we have to abandon the ARDSnet protocol (high-PEEP, low-Vt). Point-of-care echocardiography may guide decisions.

## Data Availability

Not applicable
